# Transcriptome profiling analysis of senescent gingival fibroblasts in response to *Fusobacterium nucleatum* infection

**DOI:** 10.1371/journal.pone.0188755

**Published:** 2017-11-30

**Authors:** Sun-Hee Ahn, Sung-Min Chun, Chungoo Park, Jong-Hee Lee, Seok-Woo Lee, Tae-Hoon Lee

**Affiliations:** 1 Department of Oral Biochemistry, Dental Science Research Institute, Medical Research Center for Biomineralization Disorders, School of Dentistry, Chonnam National University, Gwangju, Republic of Korea; 2 School of Biological Sciences and Technology, Chonnam National University, Gwangju, Republic of Korea; 3 World Institute of Kimchi, Gwangju, Republic of Korea; 4 Department of Dental Education and Periodontology, Dental Science Research Institute, School of Dentistry, Chonnam National University, Gwangju, Republic of Korea; 5 Department of Molecular Medicine (BK21plus), Chonnam National University Graduate School, Gwangju, Republic of Korea; J Craig Venter Institute, UNITED STATES

## Abstract

Periodontal disease is caused by dental plaque biofilms. *Fusobacterium nucleatum* is an important periodontal pathogen involved in the development of bacterial complexity in dental plaque biofilms. Human gingival fibroblasts (GFs) act as the first line of defense against oral microorganisms and locally orchestrate immune responses by triggering the production of reactive oxygen species and pro-inflammatory cytokines (IL-6 and IL-8). The frequency and severity of periodontal diseases is known to increase in elderly subjects. However, despite several studies exploring the effects of aging in periodontal disease, the underlying mechanisms through which aging affects the interaction between *F*. *nucleatum* and human GFs remain unclear. To identify genes affected by infection, aging, or both, we performed an RNA-Seq analysis using GFs isolated from a single healthy donor that were passaged for a short period of time (P4) ‘young GFs’ or for longer period of time (P22) ‘old GFs’, and infected or not with *F*. *nucleatum*. Comparing *F*. *nucleatum*-infected and uninfected GF(P4) cells the differentially expressed genes (DEGs) were involved in host defense mechanisms (i.e., immune responses and defense responses), whereas comparing *F*. *nucleatum*-infected and uninfected GF(P22) cells the DEGs were involved in cell maintenance (i.e., TGF-β signaling, skeletal development). Most DEGs in *F*. *nucleatum*-infected GF(P22) cells were downregulated (85%) and were significantly associated with host defense responses such as inflammatory responses, when compared to the DEGs in *F*. *nucleatum*-infected GF(P4) cells. Five genes (GADD45b, KLF10, CSRNP1, ID1, and TM4SF1) were upregulated in response to *F*. *nucleatum* infection; however, this effect was only seen in GF(P22) cells. The genes identified here appear to interact with each other in a network associated with free radical scavenging, cell cycle, and cancer; therefore, they could be potential candidates involved in the aged GF’s response to *F*. *nucleatum* infection. Further studies are needed to confirm these observations.

## Introduction

Periodontitis is one of the most prevalent diseases worldwide and is the major cause of tooth loss in adults. Periodontitis is a chronic, multifactorial, polymicrobial infection that is initiated by dental plaque biofilms located in the gingival sulcus, and eventually leads to gingival inflammation and alveolar bone loss [1].

*Fusobacterium nucleatum* is a facultative oral bacterium that mediates aggregations between early (streptococci and actinomycetes) and late colonizers (*Porphyromonas gingivalis*, *Tannerella forsythia*, and *Treponema denticola*), which are strongly implicated in the pathogenesis of periodontal disease [2, 3]. It is known that *F*. *nucleatum* contributes to the reducing environment necessary for the emergence of oxygen-intolerant anaerobes [4, 5]. We recently reported that *F*. *nucleatum* stimulates *P*. *gingivalis* growth by triggering the activation of NADPH oxidase in host cells, which could provide a favorable environment for strictly anaerobic bacteria [6]. Although *F*. *nucleatum* may not be directly responsible for the severe pathological damage to periodontal tissues, it is an important intermediate species, bridging the attachment of several pathogenic bacteria associated with destructive periodontal disease. Moreover, *F*. *nucleatum* adheres to, and invades, human epithelial cells directly and might mediate the entry of non-invasive bacteria.

Human gingival fibroblasts (GFs) are the major cells in periodontal tissues and play a critical role as a protective barrier against various pathogenic microorganisms [5]. Human GFs are constantly exposed to a range of different oral bacteria, triggering the production of pro-inflammatory cytokines (IL-6 and IL-8) and various metalloproteases (MMP-3, -9, and -13) [7, 8]. The secreted cytokines then recruit immune cells to combat the infection [9] whereas the MMPs are associated with wound healing and tissue remodeling [10]. The susceptibility of GFs to bacterial infection is known to be affected by aging [8, 11, 12] and furthermore, the development and progression of periodontal disease is known to be significantly associated with aging [13, 14].

Infectious diseases are among the major causes of morbidity and mortality in the elderly population. A variety of factors contribute to the increased susceptibility to infection seen in elderly people [15]. There have been many studies addressing infectious diseases in elderly populations [16–18]. For example, it is well known that elderly people are more susceptible to infection with diverse pathogens including *Streptococcus pneumoniae* [19], *Mycobacterium tuberculosis* [20], *Staphylococcus aureus* [21], and *Escherichia coli* [22]. These infections can occur at various infection sites including the respiratory tract, skin, and the urinary tract. Despite several studies regarding the effects of aging on periodontal diseases [12, 23], the underlying mechanisms through which aging affects the interaction between *F*. *nucleatum* and human GFs remain unclear.

Senescence, a process in which normal somatic cells undergo an irreversible arrest in their proliferative capacity after a limited number of divisions [24], is thought to contribute to organismal aging [24, 25]. Senescent cells are therefore a useful tool for studying aging and age-related diseases [26]. Under standard culture conditions, primary human cells undergo a limited number of cell divisions, and after several serial passages in culture, the cells enter a senescent state [24]. In general, senescent cells exhibit a complex phenotype characterized by cell cycle arrest, increased cell size, altered morphology, and altered gene expression [27]. We previously reported that aged GFs (P22) can be more easily invaded by *F*. *nucleatum*, as well as having reduced reactive oxygen species (ROS) generation and cytokine responses, compared to young GFs [8].

In this study, we aimed to investigate the differences in gene expression between young (P4) and aged (P22) GFs in response to *F*. *nucleatum* infection using a deep RNA-seq analysis (about 20 Gb per sample). The differentially expressed genes (DEGs) in young GFs (P4), most of which were upregulated by *F*. *nucleatum* infection, were involved in host defense mechanisms, such as cell death and survival-related functions. In contrast, the DEGs in aged GFs (P22) were involved in cellular maintenance such as skeletal and cellular development-related functions. *F*. *nucleatum* genes themselves were associated with more active metabolic pathways including glycolysis, fatty acid/butyrate, and cell wall synthesis, and were more highly expressed in *F*. *nucleatum*-infected young GFs (P4) compared to aged GFs (P22). By comparing the DEGs, in response to *F*. *nucleatum* infection we found that twenty-four genes were unique to young GFs (P4), whereas ten genes were unique to aged GFs (P22). Among these latter ten DEGs, five were upregulated as a result of *F*. *nucleatum* infection of aged GFs (P22) and are known to be localized in the nucleus. These five genes require further study to determine their role in aging-related GF responses to infection.

## Materials and methods

### Ethics statement

This study was approved by the Chonnam National University Dental Hospital Institutional Review Board (approval No. CNUDH-2013-001). Written informed consent was obtained for all subjects after the nature and possible consequences of the studies were explained. All participants were adults without periodontal disease.

### Cell cultures and reagents

Primary human GFs were prepared as previously described that all cells used in this study were obtained from a single healthy donor [6]. The collection of human gingival tissue was approved by the Chonnam National University IRB as described above. GFs were grown in Dulbecco's modified Eagle’s medium (DMEM; Gibco BRL, Grand Island, NY, USA) supplemented with 10% heat-inactivated fetal bovine serum (PAA Laboratories, Etobicoke, Ontario, Canada), 100 U/mL penicillin, and 100 μg/mL streptomycin (Gibco BRL) at 37°C in a humidified atmosphere containing 5% CO_2_. When confluent, the cells were trypsinized using a 0.25% trypsin/0.02% EDTA solution (Sigma, St Louis, MO, USA) and subcultured at a 1:3 ratio until the required passage number was reached and senescent characteristics were observed [8]. The cells used for all of the experiments were at either the fourth passage (P4) or the twenty-second passage (P22). Aged GFs at passage 22 were previously confirmed to have senescence-associated β-galactosidase (SA-β-gal) activity and express senescence markers such as p53, p21, and Cav-1 [8].

### Bacterial strains and culture

*F*. *nucleatum* subsp. *polymorphum* (ATCC10953) was used in this study. Bacterial cells were prepared as previously described [8]. Briefly, *F*. *nucleatum* was cultured under anaerobic condition (85% N_2_, 5% CO_2_, and 10% H_2_) at 37°C in a tryptic soy broth containing 5 μg/mL hemin (Sigma) and 1 μg/mL menadione (Sigma). Bacteria were harvested by centrifugation at 3000 rpm for 10 minutes at 4°C, washed once in phosphate-buffered saline and resuspended in DPBS. GFs were infected with *F*. *nucleatum* for 2 h at an MOI = 10.

### RNA isolation and sequencing

RNA was extracted from human GFs using the RNeasy Mini Kit (Qiagen, Hilden, Germany). The isolated RNA was treated with DNase I to remove any contaminating DNA. RNA quality was assessed using RNA screentape from the TapeStation system (Agilent Technologies, Santa Clara, CA, USA). The RIN scores for all RNA samples were higher than 7.

The mRNA-Seq sample was obtained using the Illumina TruSeq™ RNA Sample Preparation Kit (Illumina, Inc., San Diego, CA, USA). In brief, total RNA samples were treated with the RiboZero Human kit and the RiboZero bacteria kit (Epicentre) to deplete bacterial and eukaryotic rRNA, followed by thermal mRNA fragmentation. The RNA fragments were then transcribed into first strand cDNA using reverse transcriptase and random primers. The cDNA was synthesized to second strand cDNA using DNA Polymerase I and RNase H. After the end-repair process, single 'A' bases were added to the fragments and the adapters were then ligated and prepared for cDNA hybridization into the flow cell. Finally, the products were purified and enriched by PCR to create the cDNA library (Macrogen, Seoul, Korea). The cDNA libraries were sequenced on the HiSeq 2000 (Illumina) to obtain approximately 1 billion paired-end reads (2 x 101 bp).

### Transcriptome analysis

**Fig 1:**
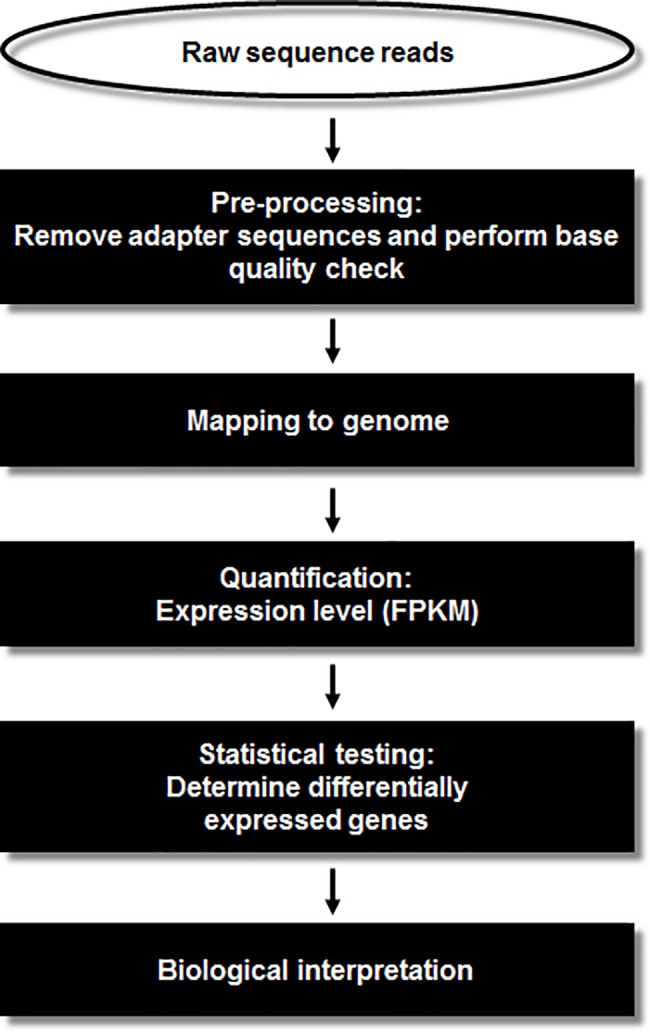
RNA-seq analysis pipeline. Laboratory pipeline for simultaneous depletion of rRNA from prokaryotic and eukaryotic RNA mixtures. The enriched mRNA was used to generate the RNA-Seq libraries.

The experimental procedures for the transcriptome analysis are illustrated in Fig 1. Initially, we pre-processed the RNA-seq data from our four samples using Trimmomatic (version 0.33) [28], to obtain clean reads by removing those containing adapter sequences, poly-N sequences, or low quality bases (below a mean Phred score of 15). The trimmed reads were aligned separately to the human and *F*. *nucleatum* genomes by Tophat2 [29] using default parameters. The genome sequences and the human (GRCh38) and *F*. *nucleatum* annotations were obtained from the NCBI genome database (https://www.ncbi.nlm.nih.gov/genome). For quantitation of mRNA transcripts, the resulting aligned reads were put into Cufflinks (v2.2.1) [29]. Unless otherwise stated, all gene expression levels used in our analyses are given using FPKM (*F*ragment *P*er *K*ilobase of exon per *M*illion fragments mapped) as the unit. Differential expression analyses were performed using Cuffdiff (v2.2.1) [29] and their visualization was generated using R (R Development Core Team, R Foundation for Statistical Computing, Vienna, Austria).

#### Gene ontology (GO), network, and pathway analyses

We performed GO and functional pathway analyses of DEGs using the GATHER tool (http://gather.genome.duke.edu/) [30, 31] and Ingenuity Pathway Analysis (IPA), version 8.0 (Ingenuity® Systems, www.ingenuity.com), respectively. To investigate the enrichment analysis of the reads of *F*. *nucleatum* metabolic pathways involved in GF aging, a Kyoto Encyclopedia of Genes and Genomes (KEGG) pathway analysis was performed [17].

## Results

### RNA-seq data analysis

**Fig 2:**
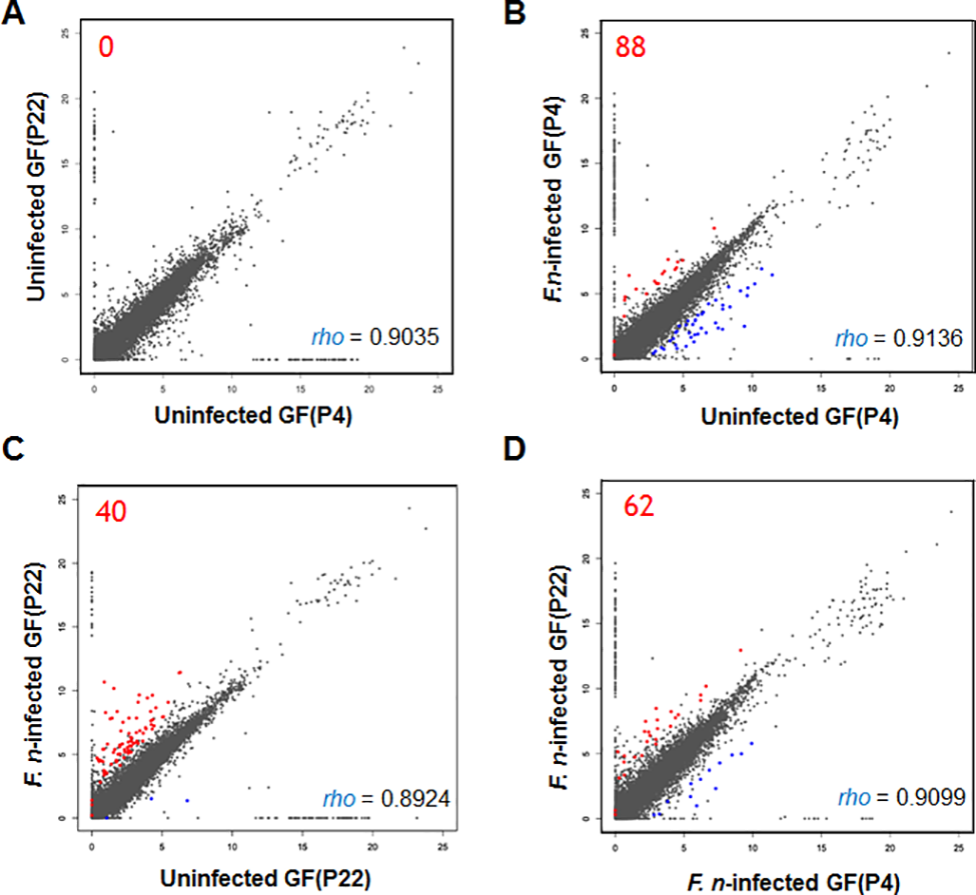
Distribution of RNA-seq data. All gene expression levels were transformed to base two logarithms. (A) Uninfected GFs (P4) vs. uninfected GFs (P22). (B) Uninfected GFs (P4) vs. *Fusobacterium nucleatum*-infected GFs (P4). (C) Uninfected GFs (P22) vs. *F*. *nucleatum*-infected GFs (P22). (D) *F*. *nucleatum*-infected GFs (P4) vs. *F*. *nucleatum*-infected GFs (P22). The individual Spearman's rank correlation coefficients are shown on each graph. Significant DEGs between the two samples, with an FDR <5% when Benjamini-Hochberg multiple testing adjustment was used, are shown in red dots (upregulated) and blue dots (downregulated).

To better understand the global responses of GFs to *F*. *nucleatum* infection and the effect of aging, we performed a genome-wide transcriptome analysis using RNA-seq technology to determine the changes in gene expression in young GFs (P4) and aged GFs (P22), with or without *F*. *nucleatum*, infection. A total of 100 Gb of raw sequence data was generated from all four samples (Table 1). After trimming the raw sequence data, the clean reads of each sample were first mapped to the human genome, and the unmapped reads in the *F*. *nucleatum*-infected samples were remapped to the *F*. *nucleatum* genome (Table 1). A total of 433,937 and 109,468 sequence reads were uniquely matched to the *F*. *nucleatum* genome in *F*. *nucleatum*-infected GF(P4) cells and *F*. *nucleatum*-infected GF(P22) cells, respectively. Their GC contents were found to be similar (35.1% and 32.7%, respectively), and were different from the percentages obtained from sequences reads following mapping onto the human reference genome (51.11% and 51.08%, respectively). These results indicated that bacterial-specific sequences, having no homology with human DNA were well achieved. The distributions of normalized FPKM values are shown in Fig 2. The distribution of gene expression is shown as scatter plots using a one to one comparison. The overall expression patterns were similar to one another (R value ranges from 0.89 to 0.91).

**Table 1:** RNA-seq data statistics.

Samples	Reference	Total reads (Raw data)	Total reads (w/o adaptor)	Aligned pairs	Multiple alignments	Concordant pair alignments
Uninfected GF(P4)	Homo sapiens	240,914,330	240,591,122	109,770,915	4,661,202	108,834,930 (90.5%)*
*F*. *nucleatum*-infected GF(P4)	Homo sapiens	249,525,110	249,311,972	107,274,408	3,153,643	106,723,775 (85.6%)
	*F*. *nucleatum* ATCC25586			473,131	39,194	433,937 (0.1741%)
Uninfected GF(P22)	Homo sapiens	266,758,386	266,438,682	124,654,404	2,965,534	123,496,658 (92.7%)
*F*. *nucleatum*-infected GF(P22)	Homo sapiens	266,580,404	259,574,938	121,806,945	4,613,353	120,318,603(92.7%)
	*F*. *nucleatum* ATCC25586			111,166	1,698	109,468(0.0422%)

Note:

* Percentage alignment of concordant pairs

### Differential expression analysis

**Fig 3:**
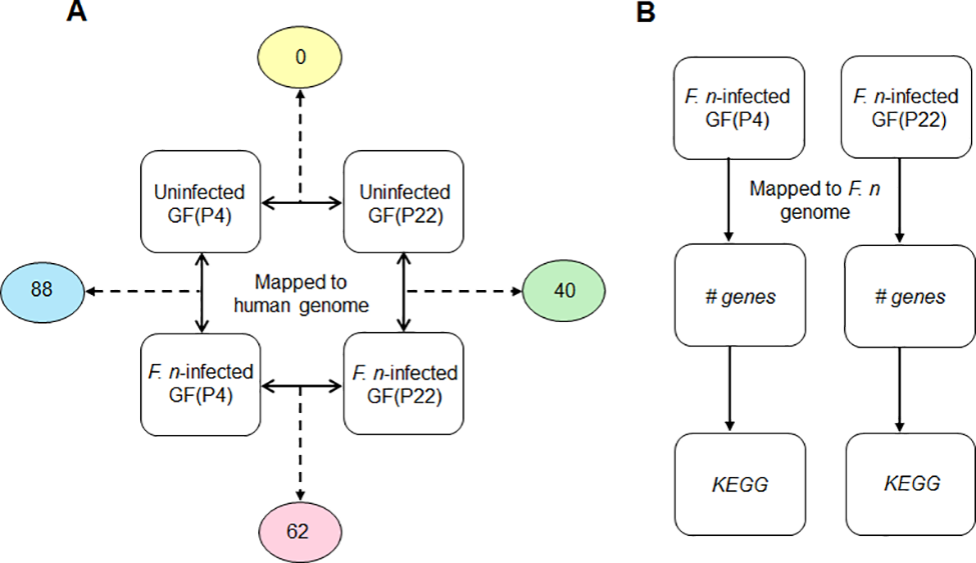
Strategy for the analysis of RNA-seq data. (A) The RNA-seq data was mapped onto the human genome and the indicated comparisons were conducted. The number of DEGs from each comparison is indicated in the circle. (B) The RNA-seq data were mapped onto the *Fusobacterium nucleatum* genome.

To investigate the age-related changes in GFs following *F*. *nucleatum* infection, the transcriptome profiles of uninfected cells versus infected cells, for both young and old cells, were compared. First, we compared the gene expression patterns between uninfected GFs and *F*. *nucleatum-*infected GFs at early (P4) and late passages (P22). From this analysis, we identified eighty-eight and forty genes that were significantly differentially expressed in *F*. *nucleatum-*infected GF(P4) and GF(P22) cells, respectively, compared to the corresponding uninfected cells (Fig 3A). These gene sets therefore represent host responses to *F*. *nucleatum* infection in young (P4) and aged (P22) GFs, respectively. We also directly compared the transcriptome profiles of both GF(P4) and GF(P22) cells following *F*. *nucleatum* infection, as well as uninfected GF(P4) and GF(P22) cells, to identify gene expression changes relating to aging following infection, or to identify gene expression changes related to aging itself. As shown in Fig 3A, we did not find any genes that were significantly altered between uninfected GF(P4) and uninfected GF(P22) cells; however, we found sixty-two genes that were significantly differentially expressed between *F*. *nucleatum*-infected GF(P4) and *F*. *nucleatum*-infected GF(P22) cells. These sixty-two genes represent aging-related changes in the host response to *F*. *nucleatum* infection.

**Fig 4:**
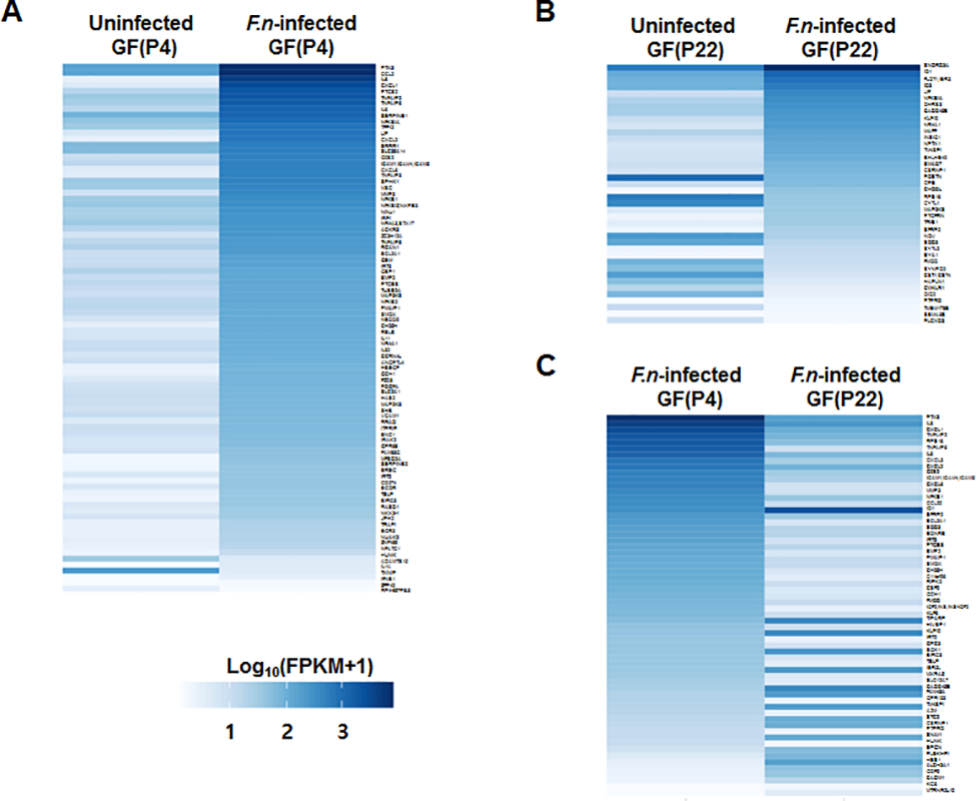
Heat maps for the different gene expression comparisons. (A) Heat map of the eighty-eight DEGs identified between uninfected GF(P4) and *F*. *nucleatum*-infected GF(P4) cells. (B) Heat map of the forty DEGs identified between uninfected GF(P22) and *Fusobacterium nucleatum*-infected GF(P22) cells. (C) Heat map of the sixty-two DEGs identified between *F*. *nucleatum*-infected GF(P4) and *F*. *nucleatum*-infected GF(P22) cells. Genes were considered to be significantly differentially expressed if they obtained a *q* < 0.05 using a Benjamini-Hochberg multiple testing adjustment.

Full lists of all of these differentially expressed genes are shown in S2, S3 and S4 Tables. Intriguingly, in GF(P4) cells, only a few genes (3%, 3 out of 88) were downregulated by infection, whereas the vast majority of the DEGs (97%, 85 out of 88) were upregulated by infection (Fig 4A). However, compared to GF(P4) cells, a relatively greater percentage of DEGs (32.5%, 13 out of 40) in GF(P22) cells were found to be downregulated by infection; conversely, 67.5% of DEGs (27 out of 40) were upregulated by infection (Fig 4B). In addition, when *F*. *nucleatum*-infected GF(P4) cells were compared with *F*. *nucleatum*-infected GF(P22) cells, 70% of the DEGs (43 out of 62) were downregulated in the *F*. *nucleatum*-infected GF(P22) cells (Fig 4C).

To investigate how *F*. *nucleatum* itself responds to the host age status, we collected all the sequence reads that were unmapped to the human genome and mapped them to the *F*. *nucleatum* genome, and in this way we identified *F*. *nucleatum* genes that were differentially expressed in GFs according to host age. A comparison of the gene expression of *F*. *nucleatum* in GF(P4) and GF(P22) cells was carried out and we found 391 and 224 genes that were highly expressed (more than a 10-fold increase) in these cells, respectively (S1 Table). We then analyzed the pathways these bacterial genes were involved in using the KEGG database. As a result, a relatively large number of the *F*. *nucleatum* genes (65 out of 391) in GF(P4) could be mapped to metabolic pathways, compared to those (11 out of 224) in GF(P22). When we compared the different bacterial pathways active in GF(P4) and GF(P22) cells, a larger variety of pathways were detected in GF(P4). For example, glycolysis, fatty acid metabolism, butyrate metabolism, and LPS/peptidoglycan biosynthesis pathways were identified as being highly expressed in *F*. *nucleatum*-infected GF(P4) cells, but none of these pathways appeared to be highly expressed in *F*. *nucleatum*-infected GF(P22) cells.

### GO enrichment analysis of DEGs

Next, to investigate the biological relevance of these DEGs, we performed a GO analysis using the GATHER database. The top five significant GO annotations for each DEG set are listed in Table 2. It is notable that the relevant GO biological processes identified in the DEGs in response to infection in GF(P4) cells were remarkably different to those GO biological processes identified in response to infection in GF(P22) cells.

**Table 2:** Top five enriched GO terms for the DEGs from each comparison.

Comparison groups	GO annotation	*p*-value*	Genes
DEGs from uninfected GF(P4) vs. *F*. *n*-infected GF(P4)	GO:0006955 [4]: immune response	<0.0001	CCL2, CXCL1, CXCL2, CXCL6, GBP1, GEM, GPR68, IFIT2, IFIT3, IFNB1, IL11, IL1A, IL32, IL6, IL8, IRAK2, IRF1, LIF, NFKB1, PDCD1LG1, PTGES, PTGS2, PTX3, TNFAIP6
	GO:0006952 [5]: defense response	<0.0001	CCL2, CXCL1, CXCL2, CXCL6, GBP1, GEM, GPR68, IFIT2, IFIT3, IFNB1, IL11, IL1A, IL32, IL6, IL8, IRAK2, IRF1, LIF, NFKB1, PDCD1LG1, PTGES, PTGS2, PTX3, TNFAIP6
	GO:0009607 [4]: response to biotic stimulus	<0.0001	CCL2, CXCL1, CXCL2, CXCL6, GBP1, GEM, GPR68, IFIT2, IFIT3, IFNB1, IL11, IL1A, IL32, IL6, IL8, IRAK2, IRF1, LIF, NFKB1, NFKBIA, PDCD1LG1, PTGES, PTGS2, PTX3, TNFAIP6
	GO:0051243 [5]: negative regulation of cellular physiological processes	<0.0001	ANGPTL4, BCL2A1, BIRC3, CCL2, CXCL1, IFNB1, IL1A, IL6, IL8, NFKB1, NFKBIA, SERPINB2, TNFAIP3, TNFAIP8
	GO:0006954 [5]: inflammatory response	<0.0001	CCL2, CXCL1, CXCL2, CXCL6, GPR68, IL1A, IL8, IRAK2, NFKB1, PTGS2, PTX3, TNFAIP6
DEGs from uninfected GF(P22) vs. *F*. *n*-infected GF(P22)	GO:0007179 [8]: transforming growth factor beta receptor signaling pathway	<0.0001	FMOD, KLF10, SMAD7
	GO:0007178 [7]: transmembrane receptor protein serine/threonine kinase signaling pathway	0.0001	FMOD, KLF10, SMAD7
	GO:0007167 [6]: enzyme-linked receptor protein signaling pathway	0.0004	FMOD, KLF10, PTPRD, SMAD7
	GO:0007275 [2]: development	0.0008	CMKLR1, EYA1, GADD45B, ID1, ID3, IER3, KLF10, LIF, NOV, NPTX1, POSTN, SEMA6B
	GO:0001501 [5]: skeletal development	0.04	CMKLR1, KLF10, POSTN
DEGs from *F*. *n*-infected GF(P4) vs. *F*. *n*-infected GF(P22)	GO:0006954 [5]: inflammatory response	<0.0001	CCL20, CXCL1, CXCL2, CXCL3, CXCL6, IL8, NFKB1, PTX3, RIPK2, TNFAIP6
	GO:0009611 [5]: response to wounding	<0.0001	CCL20, CSF2, CXCL1, CXCL2, CXCL3, CXCL6, IL8, NFKB1, PTX3, RIPK2, TNFAIP6
	GO:0006955 [4]: immune response	<0.0001	CCL20, CSF2, CXCL1, CXCL2, CXCL3, CXCL6, IFIT2, IFIT3, IL6, IL8, KLF6, NFKB1, PTGES, PTX3, RIPK2, TNFAIP6
	GO:0009613 [5]: response to pest, pathogen, or parasite	<0.0001	CCL20, CSF2, CXCL1, CXCL2, CXCL3, CXCL6, IL6, IL8, NFKB1, PTGES, PTX3, RIPK2, TNFAIP6
	GO:0043207 [5]: response to external biotic stimulus	<0.0001	CCL20, CSF2, CXCL1, CXCL2, CXCL3, CXCL6, IL6, IL8, NFKB1, PTGES, PTX3, RIPK2, TNFAIP6

*p-value was calculated using Fischer’s exact test, which calculates the ratio of the pathway-associated genes in the experimental data to the total number of genes in that pathway.

For example, the eighty-eight DEGs identified in infected GF(P4) cells were more likely to be involved in host defense to bacterial infection such as immune responses, response to biotic stimulus, and inflammatory responses. However, the forty-four DEGs in infected GF(P22) cells were highly involved in cell maintenance such as the transforming growth factor beta receptor signaling pathway and skeletal development. Moreover, the sixty-two DEGs identified between *F*. *nucleatum*-infected GF(P4) and GF(P22) cells were associated with host responses to bacterial infection such as inflammatory responses, response to wounding, and immune responses.

### Analysis of young GF(P4)- and aged GF(P22)-specific DEGs

**Fig 5:**
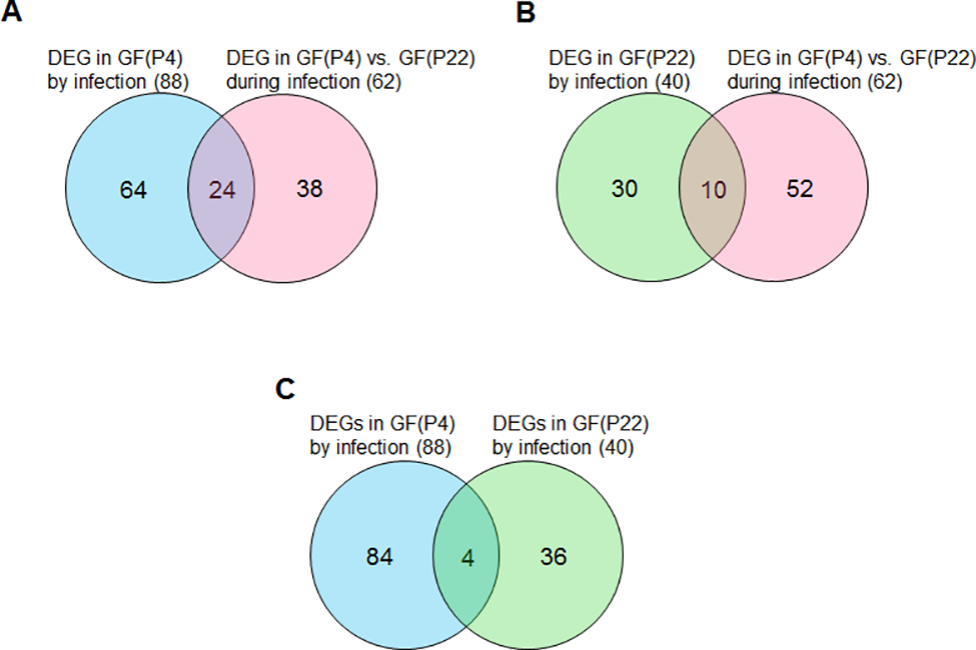
Venn diagram summarizing the overlap analysis of DEGs from the three paired comparisons. (A) Young GF(P4)-specific response to infection. (B) Aged GF(P22)-specific response to infection. (C) Common genes in young (P4) and aged (P22) GFs in response to infection.

The eighty-eight DEGs identified in *F*. *nucleatum*-infected GF(P4) cells, the forty DEGs from *F*. *nucleatum*-infected GF(22) cells, and the sixty-two DEGs found in *F*. *nucleatum*-infected GF(P4) versus GF(22) cells, were compared using an overlap analysis. As a result, we identified twenty-four DEGs that were overlapping between the eighty-eight *F*. *nucleatum*-infected GF(P4) DEGs and the sixty-two *F*. *nucleatum*-infected GF(P4) versus GF(22) DEGs. We also identified ten DEGs that were overlapping between the forty *F*. *nucleatum*-infected GF(22) DEGs and the sixty-two *F*. *nucleatum*-infected GF(P4) versus GF(22) DEGs (Fig 5A and 5B). These genes represent young GF(P4)- and aged GF(P22)-specific genes that respond to infection. Moreover, we directly compared the eighty-eight *F*. *nucleatum*-infected GF(P4) DEGs and the forty *F*. *nucleatum*-infected GF(22) DEGs, and as a result we identified only four overlapping genes (Fig 5C). These four genes reflect a common response to *F*. *nucleatum* infection between young GF(P4) and old GF(P22) cells. These overlapping gene are listed in Tables 3, 4 and 5. In addition, a heatmap showing the expression levels of these overlapping genes is shown in S1 Fig. The expression levels of both the twenty-four genes and the ten genes described above were significantly changed in GF(P4) and GF(P22), respectively, after infection with *F*. *nucleatum* (S1A and S1B Fig). The four genes described above had the same pattern of expression in both young GF(P4) and aged GF(P22) cells in response to *F*. *nucleatum* infection (S1C Fig), and these genes represent the age-independent host response to *F*. *nucleatum* infection. The differential gene expression results obtained by RNA sequencing analysis were validated by performing quantitative real-time PCR analysis using three biological replicates for each gene. S2 Fig. shows that there was a significant concordance between the RNA-seq data and the q-RT PCR data for each of the three sets of DEGs we identified, with Pearson’s correlation coefficient values ranging from *R* = 0.96~0.98 (*p*-values<0.0001).

**Table 3:** List of genes that are differentially expressed in GF(P4) cells following *Fusobacterium nucleatum* infection and are also differentially expressed between *F*. *nucleatum*-infected GF(P4) and *F*. *nucleatum*-infected GF(P22) cells.

Gene Symbol	*F*. *n*-infected GF(P4)(FPKM)	*F*. *n*-infected GF(P22)(FPKM)	Fold change	*p*-value	*q*-value	Description
IL8	1649.19	118.884	13.9	0.0001	0.023	immediate early response 5-like
PTX3	2807.05	85.6365	32.8	0.0001	0.023	pentraxin 3
CXCL1	1165.34	52.8973	22.0	0.0001	0.023	chemokine (C-X-C motif) ligand 1 (melanoma growth stimulating activity, alpha)
CXCL2	312.759	44.7769	7.0	0.0001	0.023	chemokine (C-X-C motif) ligand 2
LOC102723726,TNFAIP2	827.893	42.7072	19.4	0.0001	0.023	TNF alpha induced protein 2
IL6	567.623	36.2704	15.6	0.0001	0.023	interleukin 6
NFKB1	163.171	21.6904	7.5	0.0001	0.023	nuclear factor of kappa light polypeptide gene enhancer in B-cells 1
G0S2	233.399	16.3777	14.3	0.0001	0.023	G0/G1 switch 2
ICAM1,ICAM4,ICAM5	232.878	13.0559	17.8	0.0001	0.023	intercellular adhesion molecule
PTGES	65.8038	9.17048	7.2	0.0001	0.039	prostaglandin E synthase
PMAIP1	58.0263	6.98736	8.3	0.0002	0.048	phorbol-12-myristate-13-acetate-induced protein 1
SMOX	57.2027	6.56187	8.7	0.0001	0.039	spermine oxidase
TNFAIP6	695.135	4.63915	149.8	0.0001	0.023	TNF alpha induced protein 6
MMP3	185.183	4.15238	44.6	0.0001	0.023	matrix metallopeptidase 3
IFIT3	78.4845	4.11153	19.1	0.0001	0.023	interferon induced protein with tetratricopeptide repeats 3
CXCL6	230.687	3.81264	60.5	0.0001	0.023	chemokine (C-X-C motif) ligand 6
BIRC3	20.9153	3.06231	6.8	0.0001	0.023	baculoviral IAP repeat containing 3
BCL2A1	100.231	2.99886	33.4	0.0001	0.023	BCL2-related protein A1
GCH1	41.9286	2.97619	14.1	0.0002	0.048	GTP cyclohydrolase 1
BMP2	65.7944	2.7264	24.1	0.0001	0.023	bone morphogenetic protein 2
CH25H	55.7454	2.58238	21.6	0.0001	0.039	cholesterol 25-hydroxylase
TSLP	20.9051	1.09448	19.1	0.0002	0.048	thymic stromal lymphopoietin
IFIT2	22.3563	0.731211	30.6	0.0001	0.023	interferon induced protein with tetratricopeptide repeats 2
HUNK	6.15449	0.343262	17.9	0.0001	0.023	hormonally upregulated Neu-associated kinase

**Table 4:** List of genes that are differentially expressed in GF(P22) cells following *Fusobacterium nucleatum* infection and are also differentially expressed between *F*. *nucleatum*-infected GF(P4) and *F*. *nucleatum*-infected GF(P22) cells.

Gene Symbol	*F*. *n*-infected GF(P4)(FPKM)	*F*. *n*-infected GF(P22)(FPKM)	Fold change	*p*-value	*q*-value	Description
ID1	150.823	1044.03	6.9	0.00005	0.023	inhibitor of DNA binding 1, dominant negative helix-loop-helix protein
GADD45B	14.0317	196.617	14.0	0.00005	0.023	growth arrest and DNA damage inducible beta
KLF10	23.1319	172.259	7.4	0.00005	0.023	Kruppel-like factor 10
TM4SF1	10.2752	91.4458	8.9	0.00005	0.023	transmembrane 4 L six family member 1
CSRNP1	7.34259	54.0771	7.4	0.00005	0.023	cysteine-serine-rich nuclear protein 1
RPS16	798.429	27.7512	0.0	0.00005	0.023	ribosomal protein S16
SFRP2	113.416	17.6024	0.2	0.00015	0.048	secreted frizzled-related protein 2
SOD3	87.2911	10.8697	0.1	0.00005	0.023	superoxide dismutase 3, extracellular
PTPRD	6.89248	0.499976	0.1	0.00005	0.023	protein tyrosine phosphatase, receptor type, D
FMOD	39.7058	6.48497	0.2	0.00015	0.048	fibromodulin

**Table 5:** List of genes that are differentially expressed in GF(P4) cells following *Fusobacterium nucleatum* infection and in GF(P22) cells following *F*. *nucleatum* infection.

Gene Symbol	Description	Uninfected GF(P4) (FPKM)	*F*. *nucleatum* -infected GF(P4) (FPKM)	Fold change	*p*-value	*q*-value	Uninfected GF(P22) (FPKM)	*F*. *nucleatum* -infected GF(P22) (FPKM)	Fold change	*p*-value	*q*-value
LIF	leukemia inhibitory factor	2.4761	328.352	132.6	0.0001	0.012	6.85492	355.645	51.9	5E-05	0.030
NFKBIA	nuclear factor of kappa light polypeptide gene enhancer in B-cells inhibitor, alpha	22.5946	402.558	17.8	0.0001	0.012	15.7592	299.9	19.0	5E-05	0.030
MAP3K8	mitogen-activated protein kinase kinase kinase 8	5.01763	61.4196	12.2	0.0001	0.012	2.81459	27.6778	9.8	5E-05	0.030
NR4A1	nuclear receptor subfamily 4 group A member 1	5.52154	49.976	9.1	0.0001	0.012	3.62807	176.594	48.7	5E-05	0.030

### Functional annotation of DEGs by each comparison

To investigate the biological relevance of the gene sets described above, we functionally annotated the DEGs using the knowledge database IPA. The top 10 detailed categories of each set of DEGs presented in S2, S3, S4 and Tables and Tables 3 and 4 are listed in Table S5.the data showed a similar pattern as the GO analysis. For example, the biological functions of DEGs identified in young GF(P4) cells in response to infection (cell death and survival-related functions such as apoptosis, cell death, and proliferation) were distinctly different from the biological functions of DEGs identified in aged GF(P22) in response to infection (skeletal and cellular development-related functions such as morphology of skeleton, differentiation of connective tissue cells, and abnormal morphology of skeleton). Moreover, the DEGs identified using a direct comparison between *F*. *nucleatum*-infected young GF(P4) and *F*. *nucleatum*-infected aged GF(P22) cells revealed genes that are involved in cell death and survival-related functions such as apoptosis, cell death, and necrosis. In a similar context, young *F*. *nucleatum*-infected GF(P4)-specific DEGs were involved in immune responses such as esophagitis, osteoarthritis, and inflammatory responses, whereas aged *F*. *nucleatum*-infected GF(P22)-specific DEGs were associated with connective tissue disorders or developmental processes such as abnormal morphology of the skeleton and abnormal morphology of limb bones.

### Network predicted by IPA

**Fig 6:**
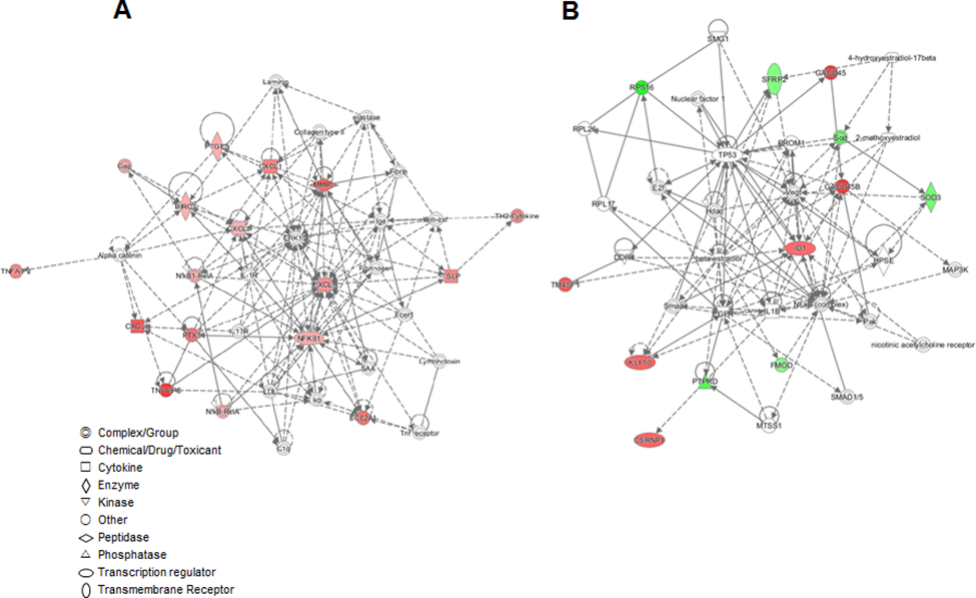
Networks predicted by IPA for the DEGs. IPA network analysis of the genes from (A) young GF(P4)-specific response to *F*. *nucleatum* infection and the genes from (B) aged GF(P22)-specific response to *F*. *nucleatum* infection. The network is displayed graphically as nodes (genes). The node color intensity indicates the expression of genes, with red representing upregulation and green representing downregulation. Solid lines and dotted lines indicate direct relationship and indirect relationships, respectively. Red and blue denote upregulated and downregulated DEGs, respectively.

To investigate the possible interactions between these differentially regulated genes, a network analysis of *F*. *nucleatum*-infected young GF(P4)-specific and *F*. *nucleatum*-infected aged GF(P22)-specific DEGs was performed using IPA. The most significant molecular networks are shown in Fig 6A and 6B. The *F*. *nucleatum*-infected young GF(P4)-specific DEGs were highly associated with networks for gastrointestinal disease, inflammatory disease, organismal injury, and abnormalities pathways (score 33 and focus molecules 13). All of these DEGs were upregulated in *F*. *nucleatum*-infected GF(P4) cells, when compared to *F*. *nucleatum*-infected GF(P22) cells, and the majority of genes were related to NF-kB activation and various chemokines. Moreover, *F*. *nucleatum*-infected GF(P22)-specific DEGs were highly associated with networks for free radical scavenging, cell cycle, and cancer (score 30 and focus molecules 13). One gene involved in free radical scavenging, SOD3, was downregulated, whereas the genes associated with cell cycle or cancer, including GADD45B, ID1, KLF10, CSRNP1, and TM4SF1, were upregulated in *F*. *nucleatum*-infected GF(P22) cells when compared to *F*. *nucleatum*-infected GF(P4) cells. It is notable that all the upregulated DEGs in *F*. *nucleatum*-infected GF(P4) cells were localized in the nucleus and cytoplasm. Although the upregulated genes in *F*. *nucleatum*-infected GF(P22) cells were also localized in the nucleus, the downregulated genes were localized in the cytoplasm (S3 Fig).

## Discussion

Using RNA-seq technology, this study reports a novel, quantitative, and comprehensive gene expression mapping in GFs following *F*. *nucleatum* infection, and furthermore examines the effect of cell age. In previous studies, we have shown that *F*. *nucleatum* infection in GFs triggers ROS generation, which is involved in the host defense mechanism. This activation of NADPH oxidase occurs 2 h post-infection with *F*. *nucleatum* [6], and was reduced in aged GF(P22) cells [8]. In the present study, we used an RNA-seq strategy to assess the overall impact of aging on the host response to *F*. *nucleatum* at an early stage of infection (2 h), which exhibiting bacterial invasion and host defense mechanisms, according to previous studies [6, 8]. We also attempted to determine changes in the *F*. *nucleatum* gene expression pattern between old and young infected GFs using the same RNA-seq technology.

RNA-seq has been widely used in many differential gene expression studies [32, 33]. It is a comprehensive and systematic approach to defining the transcriptome of an organism with minimal bias [34], that can be used across various cell types and experimental settings [35, 36], without specific probes or cross-hybridization issues. However, parallel RNA-Seq profiling of both prokaryotic and eukaryotic gene expression in bacterially-infected cells is technically challenging. Total RNA extracted from bacterially-infected mammalian cells is a heterogeneous mixture of host and bacterial RNAs. Ribosomal RNA (rRNA) is the most abundant RNA in the cell (accounting for up to 98% of total RNA) [34]. Bacterial mRNA is typically a minor portion of the total RNA in an infected host cell. To approach bacterial RNA sequencing, many studies have tried to directly isolate bacterial mRNA from eukaryotic mRNA to then amplify it because it is present at extremely low levels. However, this could result in unnecessary loss and over interpretation by RNA amplification of small amounts. Thus, in this study we favored a deep RNA-seq (20 Gb depth) approach, rather than the typical depth for eukaryotic RNA-seq of 6 Gb, in order to be able to obtain reads for bacterial mRNAs. As a result, when we mapped the RNA-seq data onto the *F*. *nucleatum* genome sequence, almost 70% of the open reading frames appeared from the total RNA-seq data. By comparing bacterial gene expression between *F*. *nucleatum*-infected young (P4) and aged (P22) GFs, we identified 391 *F*. *nucleatum* genes that were highly expressed specifically in infected GF(P4) cells when compared to *F*. *nucleatum*-infected GF(P22) cells. In contrast, 224 *F*. *nucleatum* genes were highly expressed in infected GF(P22) cells, when compared to infected GF(P4) cells. Interestingly, the *F*. *nucleatum* genes that were highly expressed in GF(P4) cells were involved in numerous metabolic pathways including glycolysis, lipid metabolism, and biosynthesis of cell wall components. These data indicate that, during *F*. *nucleatum* infection, the host immune response predominates in young GF(P4) cells, but not in aged GF(P22) cells.

GFs act as the first physical line of defense against oral microflora and locally orchestrate immune reactions following specific recognition of pathogen-associated molecular patterns by their respective TOLL-like receptors (TLRs) [37]. *F*. *nucleatum* is considered to be more of an opportunistic pathogen that may participate in the disease process when environmental conditions allow it. From our RNA-seq data, it is notable that, among the more than 38,000 genes tested, IL-8 expression was the most significantly increased gene (about 1900-fold upregulation) in young GFs following *F*. *nucleatum* infection. IL-8 is a key chemokine for the accumulation of neutrophils. A similar upregulation of IL-8 has been found in epithelial cells infected with *H*. *pylori* [38] suggesting that it is of paramount importance in the acute inflammatory response following *H*. *pylori* infection. Several other groups have also demonstrated an increase in IL-8 in response to *H*. *pylori* infection both *in vivo* [39], and *in vitro* [40]. These data are therefore consistent with our results in *F*. *nucleatum*-infected young GFs. As expected from our previous study [8], the levels of IL-8 and IL-6 were significantly decreased in *F*. *nucleatum*-infected aged GF(P22) cells compared to that in *F*. *nucleatum*-infected young GF(P4) cells (S4 Table). According to Eftang et al., the increase in IL-8 in *H*. *pylori*-infected gastric epithelial cells can be explained by the upregulation of NF-kB, TNFAIP3, RELB, and BIRC3 [38]. We also found that these same four genes were upregulated in *F*. *nucleatum*-infected young GF(P4) cells (S2 Table), but not in *F*. *nucleatum*-infected aged GF(P22) cells (S3 Table).

One representative antioxidant enzyme, SOD3, was found to be downregulated in *F*. *nucleatum*-infected aged GF(P22) cells compared to *F*. *nucleatum*-infected young GF(P4) cells (Table 4 and S4 Table). SOD catalyzes the dismutation of two superoxide anion radicals into superoxide and hydrogen peroxide, which can then be removed by the actions of catalase, glutathione peroxidases, and peroxidases [41]. Three types of SOD exist in cells, Cu, Zn-SOD (SOD1) in the cytosol, and Mn-SOD (SOD2) in mitochondria. The third form, also containing Cu and Zn (SOD3), is found extracellularly. There have been numerous studies examining changes in SOD activity with aging, but the results have been inconsistent. It has also been reported that there is an increase in SOD3 with aging in the prostatic lobes [42] and renal cortex of rats [43]. In contrast, SOD3 expression has been reported to be decreased in retinal pigment epithelial cells from older donors compared to those from younger donors [44]. Similarly, lipopolysaccharide (LPS)-treated mice showed an age-associated decrease in the expression of SOD3. Although the data in the literature are inconsistent, the latter two studies do support our data.

To the best of our knowledge, this is the first study that has used RNA-seq and IPA to assess the effect of aging and infection on the transcriptome of primary GFs. The IPA network analysis revealed that infection induced aged GF(P22)-specific DEGs were connected to each other (Fig 6) and the upregulated genes (Id1, KLF10, GADD45b, and CSRNP1) were all mainly localized in the nucleus (S3 Fig). These nuclear genes might be involved in mediating the downregulation of other target genes in aged GF(P22) cells during *F*. *nucleatum* infection.

Id1 is known to play a role in the control of senescence *in vitro*. In fact, Swarbrick et al. have also reported that overexpression of Id1 regulates senescence *in vivo* [45]. Id family proteins (Id1, Id2, Id3, and Id4) have been implicated in a variety of biological processes including cellular growth, senescence, differentiation, apoptosis, angiogenesis, and T-cell receptor signaling [46], although the role of Id family members in the regulation of these functions and the exact mechanisms are still under active investigation. The aging-related host responses that have been described so far for Id1 raise questions about its role in periodontal diseases, but currently very little is known.

KLF10 is a TGF-β responsive gene that plays a role in human osteoblasts [47]. Using knockout mice, Subramaniam et al., have described a critical role for KLF10 in osteoblast-mediated mineralization, as well as osteoblast support of osteoclast differentiation [48]. Moreover, KLF10 has been shown to have a role as either a transcriptional activator or suppressor, depending on the cell line examined [49].

GADD45B is a member of a group of genes that are usually upregulated in response to stressful growth arrest or DNA damage [50], and it has also been reported that GADD45B has pro-apoptotic activity [51]. GADD45B is therefore associated with many processes during cellular adaptation to a diverse array of cellular stresses including apoptosis, DNA repair, and cell cycle delay [52]. Chen et al., have suggested that the role of GADD45B in cell stress responses is complex, and that it can exert either protective or deleterious effects depending on the type of cell and the insult [52].

Using multiple computational tools, a prioritized list of twenty-one candidate genes involved in periodontitis has recently been reported. Among these promising genes, involved, or potentially involved, in periodontitis CXCL1 and MMP3 were also identified in our present study as being gene induced in young GFs in response to *F*. *nucleatum* infection. In contrast, the roles of GADD45B and BIRC3 have not been thoroughly investigated in the progression of periodontitis [53]. In our study, GADD45B was identified as being one of the upregulated genes in aged GF(P22) cells compared to that in young GF(P4) cells in a setting of *F*. *nucleatum* infection. It would be interesting to further investigate the role of GADD45b in the development of periodontitis in elderly subjects.

The current study has several limitations. First, this study contains a low number of biological replicates. However, the RNA-seq analysis was performed in combination with deep sequencing (20 Gb). Other studies support our approach by suggesting that most RNA-seq studies have high technical reproducibility means that a large number of technical replicates is not necessary [54], but this fact does not improve the need for biological replicates in order to make statistical inferences [55]. Moreover, large-scale RNA-seq studies with extensive differential expression analyses have frequently used limited biological replicas, favoring in its place a strategy of a low number of biological replicas coupled with deep sequencing [56, 57]. Nevertheless, to minimize the concern about biological replicates, we validated the differential expression of several gene sets using a q-PCR assay. Second, this study was based on an *in vitro* model. Several studies have been performed using *in vivo* samples, such as gingival tissue from patient with periodontitis, or aged patients [7, 58, 59]. In our previous study in which we used RNA-seq to analyze gingival tissue from young and aged subjects with no periodontal disease we found a major difference in matrix metalloprotease (MMP) expression in aged gingiva [7]. Although this result might provide a potential molecular target involved in gingival aging, it does not explain why aged patients are more susceptible to bacterial infection. Moreover, the gingival tissue used in that study contained many different cell types (i.e. it was heterogeneous), and it is likely that the natural aging process itself increases the likelihood of the gingival tissue being exposed to a number of external stimulants such as physical stress, bacterial contaminants, as well as other contaminants, thus inhibiting optimal analysis. Therefore, in the present study, we elected to focus on a specific cell type and used an *in vitro* model employing aging primary GFs.

Nevertheless, the results of this study suggest that the potential target genes identified here, especially the five genes upregulated in GF(P22) cells during *F*. *nucleatum* infection, might contribute to the aged GF(P22) response to *F*. *nucleatum* infection, which could leave aged GF(P22) susceptible to infection. In addition, we also attempted to investigate the pattern of bacterial gene expression within host cells. Taken together, our study provides important insights into the transcriptome profiling of GFs in response to *F*. *nucleatum* infection. Further investigation to elucidate the function of target genes in aged GFs will contribute to a better understanding of the mechanism by which aged cells behave following bacterial infection. In addition, these target genes might serve as potential markers for aging-related periodontal diseases.

## Supporting information

S1 Fig Heat maps of DEGs. (A) Heat map of the twenty-four DEGs that were overlapping between the eighty-eight *F*. *nucleatum*-infected GF(P4) DEGs and the sixty-two *F*. *nucleatum*-infected GF(P4) versus GF(22) DEGs (B) Heat map of the ten DEGs that were overlapping between the forty *F*. *nucleatum*-infected GF(22) DEGs and the sixty-two *F*. *nucleatum*-infected GF(P4) versus GF(22) DEGs (C) Heat map of the four genes that were overlapping between eighty-eight *F*. *nucleatum*-infected GF(P4) DEGs and the forty *F*. *nucleatum*-infected GF(22) DEGs.
(TIF)

S2 Fig Validation of the number of biological replicates used for DEG analysis by quantitative real-time PCR analysis. The expression of selected genes from the RNA sequencing data was validated by real-time PCR analysis. The x-axis represents log2 (fold change) obtained by RNA sequencing and the y-axis indicates the–ΔΔCt values. The linear regression was performed using Pearson’s correlation (*R*) and the corresponding *p* value is based in the gene expression values by both methods. (A) Twenty-eight genes upregulated in GF(P4) cells in response to *F*. *nucleatum* infection (B) Fourteen up- or down-regulated genes in GF(P22) cells in response to *F*. *nucleatum* infection, and (C) the four genes found in common in GF(P4) and GF(P22) in response to *F*. *nucleatum* infection.
(TIF)

S3 Fig Subcellular networks in the DEGs predicted by IPA. (A) Subcellular network analysis of the genes from (A) young GF(P4)-specific response to *F*. *nucleatum* infection and (B) aged GF(P22)-specific response to *F*. *nucleatum* infection.
(TIF)

S1 Table *F*. *nucleatum* genes upregulated in GF(P4) or GF(P22) cells. The reads (FPKM) for *F*. *nucleatum* were compared between infected GF(P4) and GF(P22) cells.
(XLS)

S2 Table DEGs identified between uninfected GF(P4) and *F*. *nucleatum*-infected GF(P4) cells. (XLS)

S3 Table DEGs identified between uninfected GF(P22) and *F*. *nucleatum*-infected GF(P22) cells. (XLS)

S4 Table DEGs identified between *F*. *n*-infected GF(P4) and *F*. *nucleatum*-infected GF(P22) cells. (XLS)

S5 Table Summary of the top five functional annotations of DEGs from each comparison. All DEG datasets were analyzed using IPA software. The significance value associated with a function in Global Analysis is a measure of the likelihood that a gene from the dataset file under investigation participates in that function. The significance is expressed as a p-value, which is calculated using a right-tailed Fisher's Exact Test.
(XLS)
