# Correction: Transcriptome profiling analysis of senescent gingival fibroblasts in response to *Fusobacterium nucleatum* infection

**DOI:** 10.1371/journal.pone.0191209

**Published:** 2018-01-09

**Authors:** Sun-Hee Ahn, Seongmin Cheon, Chungoo Park, Jong-Hee Lee, Seok-Woo Lee, Tae-Hoon Lee

The second author’s name is spelled incorrectly. The correct name is: Seongmin Cheon. The correct citation is: Ahn S-H, Cheon S, Park C, Lee J-H, Lee S-W, Lee T-H (2017) Transcriptome profiling analysis of senescent gingival fibroblasts in response to *Fusobacterium nucleatum* infection. PLoS ONE 12(11): e0188755. https://doi.org/10.1371/journal.pone.0188755
