# Supplementary figures and images for: Transcriptome profiling analysis of senescent gingival fibroblasts in response to Fusobacterium nucleatum infection
Source: PLoS One. 2017 Nov 30;12(11):e0188755. doi: 10.1371/journal.pone.0188755 (PMC5708803; doi:10.1371/journal.pone.0188755)

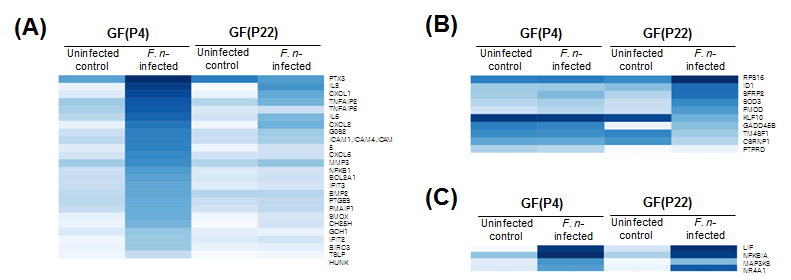

Supplement: S1 Fig — (A) Heat map of the twenty-four DEGs that were overlapping between the eighty-eight F. nucleatum-infected GF(P4) DEGs and the sixty-two F. nucleatum-infected GF(P4) versus GF(22) DEGs (B) Heat map of the ten DEGs that were overlapping between the forty F. nucleatum-infected GF(22) DEGs and the sixty-two F. nucleatum-infected GF(P4) versus GF(22) DEGs (C) Heat map of the four genes that were overlapping between eighty-eight F. nucleatum-infected GF(P4) DEGs and the forty F. nucleatum-infected GF(22) DEGs. (TIF) [file pone.0188755.s001.tif]

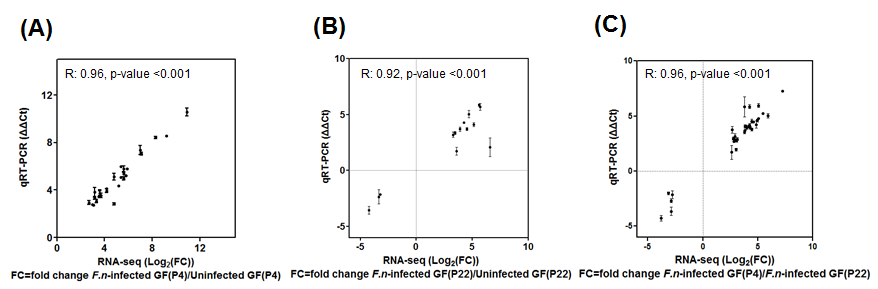

Supplement: S2 Fig — The expression of selected genes from the RNA sequencing data was validated by real-time PCR analysis. The x-axis represents log2 (fold change) obtained by RNA sequencing and the y-axis indicates the–ΔΔCt values. The linear regression was performed using Pearson’s correlation (R) and the corresponding p value is based in the gene expression values by both methods. (A) Twenty-eight genes upregulated in GF(P4) cells in response to F. nucleatum infection (B) Fourteen up- or down-regulated genes in GF(P22) cells in response to F. nucleatum infection, and (C) the four genes found in common in GF(P4) and GF(P22) in response to F. nucleatum infection. (TIF) [file pone.0188755.s002.tif]

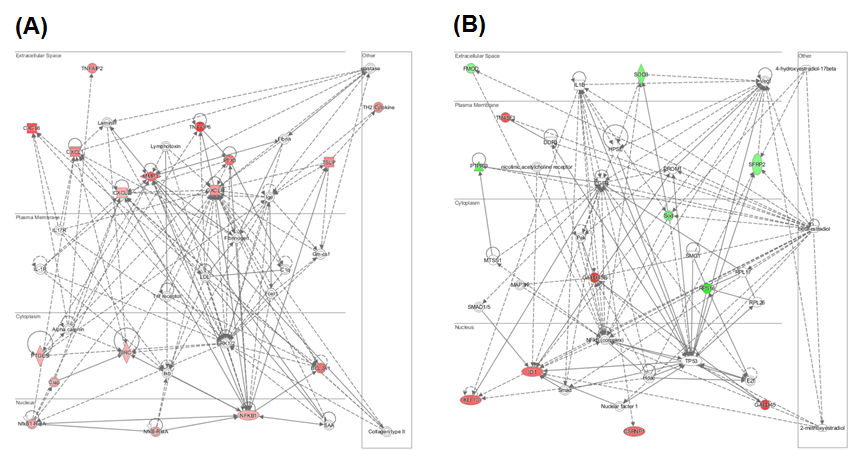

Supplement: S3 Fig — (A) Subcellular network analysis of the genes from (A) young GF(P4)-specific response to F. nucleatum infection and (B) aged GF(P22)-specific response to F. nucleatum infection. (TIF) [file pone.0188755.s003.tif]
